# Direct Arylation in the Presence of Palladium Pincer Complexes

**DOI:** 10.3390/molecules26144385

**Published:** 2021-07-20

**Authors:** Garazi Urgoitia, Maria Teresa Herrero, Fátima Churruca, Nerea Conde, Raul SanMartin

**Affiliations:** Department of Organic and Inorganic Chemistry, Faculty of Science and Technology, University of the Basque Country (UPV/EHU), 48940 Leioa, Spain; garazi.urgoitia@ehu.eus (G.U.); mariateresa.herrero@ehu.eus (M.T.H.); fatima.churruca@ehu.eus (F.C.); nerea.conde@ehu.eus (N.C.)

**Keywords:** direct arylation, palladium, pincer complexes

## Abstract

Direct arylation is an atom-economical alternative to more established procedures such as Stille, Suzuki or Negishi arylation reactions. In comparison with other palladium sources and ligands, the use of palladium pincer complexes as catalysts or pre-catalysts for direct arylation has resulted in improved efficiency, higher reaction yields, and advantageous reaction conditions. In addition to a revision of the literature concerning intra- and intermolecular direct arylation reactions performed in the presence of palladium pincer complexes, the role of these remarkably active catalysts will also be discussed.

## 1. Introduction

**Figure 1:**
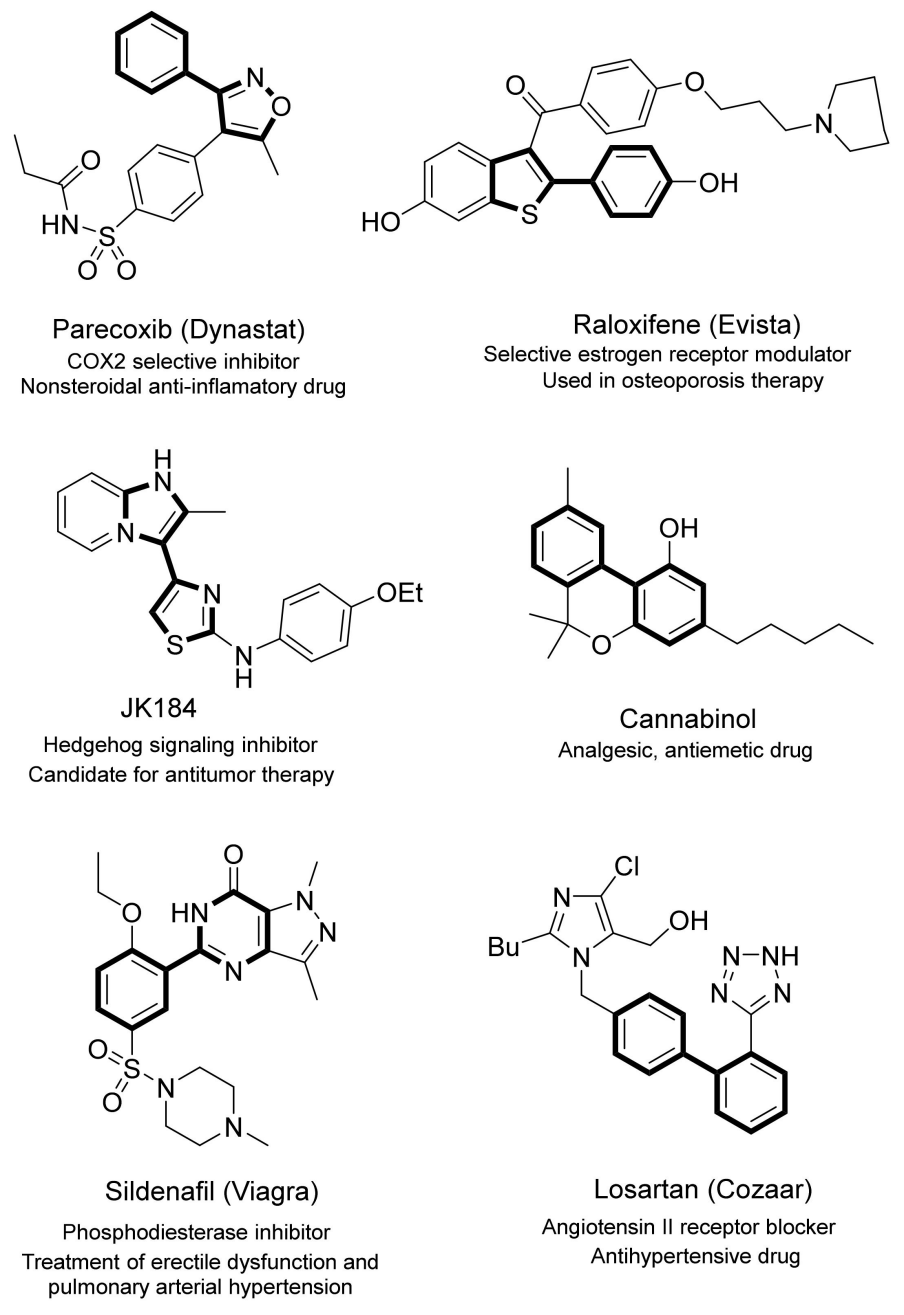
Examples of important bi(hetero)aryl-containing compounds.

Many biologically active compounds contain (hetero)biaryl frameworks. In fact, this pharmacophore core is found in a number of currently prescribed or clinically tested drugs, including several employed in the therapy against cancer or infertility, or antifungal, anti-inflammatory, anti-hypertensive and antibiotic drugs, inter alia (Figure 1) [1,2,3,4,5,6]. In addition to some agrochemical compounds, relevant materials such as liquid crystal displays and molecular switches comprise the (hetero)biaryl motif [7,8,9,10]. Among the methods developed for the preparation of (hetero)biaryl compounds, Ullmann, Scholl, and Gomberg–Bachmann reactions are considered to be classical strategies [11,12,13], whereas palladium or nickel-catalyzed cross-coupling reactions (Suzuki–Miyaura [14], Kumada [15], Stille [16] and Negishi couplings [17]) were discovered at the end of the 20th century and have been extensively utilized due to the large substrate scope and milder conditions involved. Nevertheless, pre-activated or functionalized coupling partners are required for the latter cross-coupling reactions, as (hetero)aryl halides or pseudohalides are coupled with organometallic reagents (organoboron, organomagnesium, organotin, organozinc compounds, respectively). Additional synthetic steps are therefore needed, and the coupling reaction itself often involves the generation of stoichiometric amounts of metal waste. In order to avoid these inconveniences, new methods for (hetero)aryl-aryl bond formation have been devised [18,19,20,21,22,23,24]. In this regard, direct arylation reactions have arisen as a promising alternative to the above cross-coupling strategies. Different names have been applied to define the coupling of a simple (hetero)arene with an aryl halide or pseudohalide. Among them, C–H (bond) activation, cross-dehalogenative coupling, C–H (bond) functionalization, and catalytic direct arylation are generally employed. Although pioneering reports on the use of alternative metals for direct arylation have been published (Cu [25], Fe [26], Ni [27], Ir [28] or Co [29]), second-row transition metals in low oxidation states (Rh [30,31,32,33], Ru [34,35,36,37,38], and especially Pd [39,40,41,42,43]) are preferred as catalysts for these cross-dehalogenative couplings. 

The ligands required usually depend on the nature of the haloarene coupling partner. Thus, monodentate triaryl phosphines (e.g., PPh_3_ and P(*o*-Tol)_3_) are typically used for more reactive iodoarenes. Arylation with bromo(hetero)arenes can be also carried out with the same phosphines, although with some substrates better results have been obtained using sterically crowded, electron-rich trialkylphosphanes and biphenylphosphanes [44,45,46,47,48,49,50,51]. The use of chloroarenes in most cross-coupling reactions is often hampered by the more difficult oxidative addition step [52,53]. Therefore, the palladium-catalyzed direct arylation of chloroarenes is usually carried out in the presence of the above trialkyl- and biphenylphosphanes or *N*-heterocyclic carbenes (NHC) as ligands. Jeffery’s ligand-free conditions have also been successfully used in this field [54,55,56,57,58,59,60,61]. Catalyst loading generally ranges from 1 to 20 mol%.

Alkali carbonates (K_2_CO_3_, Cs_2_CO_3_), carboxylates (KOAc, CsOPiv) and *^t^*BuOK are the bases which are usually employed, although in some cases, bases such as DBU and Et_3_N have been described. In addition to regenerate the active catalyst, it has been proposed that those bases take part in the formation of diarylpalladium(II) species [62,63,64]. In part due to the higher solubility in organic solvents, Cs_2_CO_3_ and CsOPiv have provided better results in some cases. As for solvents, although non-polar toluene and xylene have been employed, *N*,*N*-dimethylformamide (DMF), *N*,*N*-dimethylacetamide (DMA), acetonitrile, *N*-methylpyrrolidone (NMP) and dimethylsulfoxide (DMSO) are the commonly used polar aprotic solvents. Heating at temperatures ranging from 100 °C to 140 °C for several hours or days is generally required [65]. Interestingly, a recent report by Albéniz and co-workers demonstrated the beneficial and non-innocent role of alternative solvents such as pinacolone [66].

**Scheme 1:**
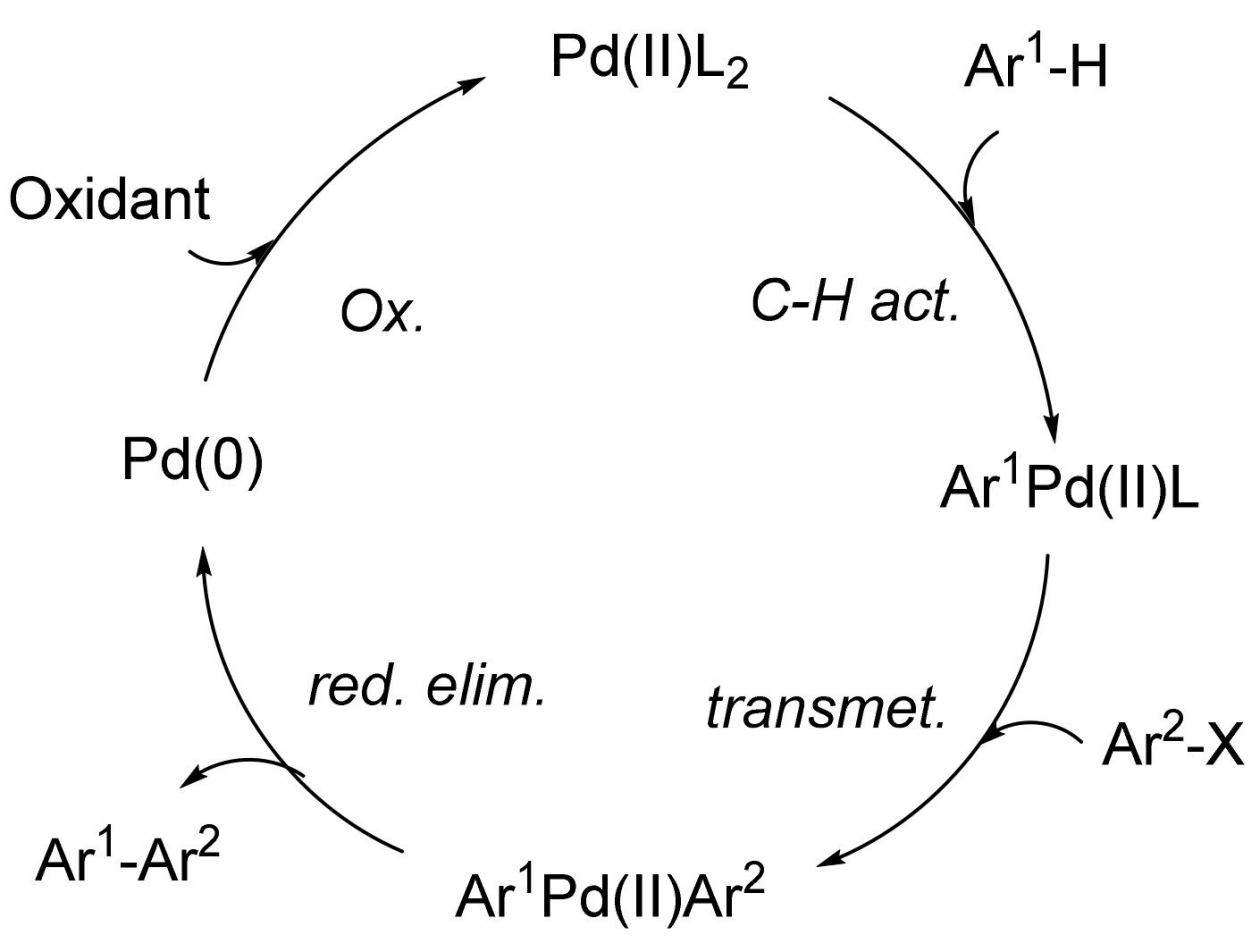
Alternative pathway to explain palladium-catalyzed direct arylation.

Several mechanisms have been proposed to explain the direct arylation process. After an initial oxidative addition step, the postulated mechanisms diverge in different pathways. Thus, an electrophilic aromatic substitution-type process might take place [67,68,69], or a concerted termolecular electrophilic substitution [70], or base-assisted intramolecular electrophilic-type substitution [71], or a *σ*-bond metathesis [72,73], a single electron transfer (SET) [74], or a carbometallation process followed by a *β*-hydride elimination [75,76], a concerted metalation deprotonation (CMD) [77], or a C–H bond oxidative addition [78,79,80]. Alternatively, in concordance with ruthenium-catalyzed arylations [81,82], a mechanistic pathway based on an initial palladium-catalyzed C–H bond activation has been proposed. As shown in Scheme 1, interaction between the Pd(II) complex and the arene would result in the generation of arylpalladium complex Ar^1^Pd(II)L, which, upon transmetallation with Ar^2^X, forms intermediate Ar^1^Pd(II)Ar^2^. After reductive elimination of the latter complex with the release of Pd(0) species and Ar^1^-Ar^2^, the catalyst would be regenerated by oxidation to Pd(II) [83].

**Scheme 2:**
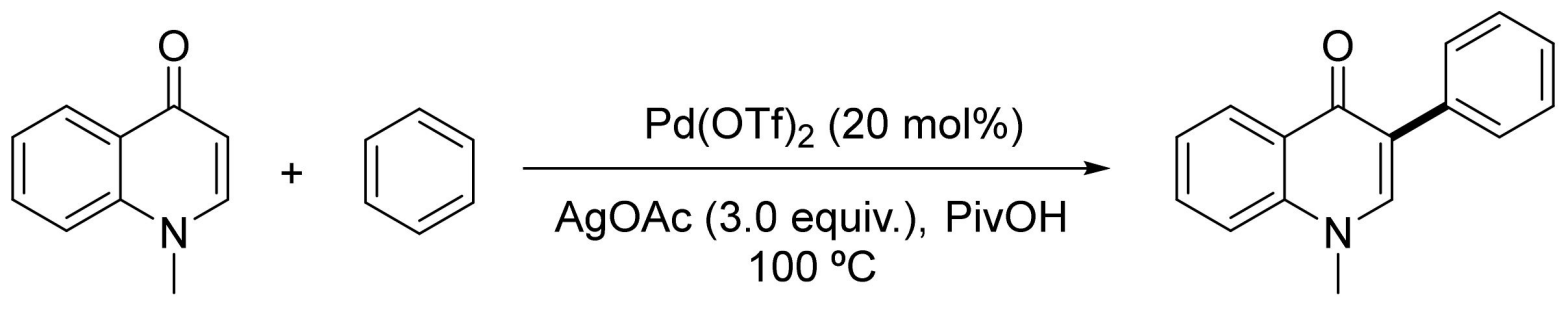
Regioselective C-3 phenylation of 1-methylquinolin-4(1H)-one.

Regioselectivity is often controlled by the electronics of the arene in which C–H functionalization takes place, by the relative C–H acidity, and by the presence of directing groups (nitrogen- or oxygen-coordinating groups, tethering groups, or intramolecular arylations) [84]. As examples of regioselective direct arylation based on the presence of directing groups, Kim and co-workers described the palladium-catalyzed C-8 arylation of dihydroisoquinolones [85]. A regioselective C-3 phenylation of 1-methylquinolin-4(1*H*)-one was reported by Choi et al. (Scheme 2) [86], and Hartwig’s group presented the regioselective arylation of a number of mono- and disubstituted arenes using synergistic silver and palladium catalysis [87].

**Figure 2:**
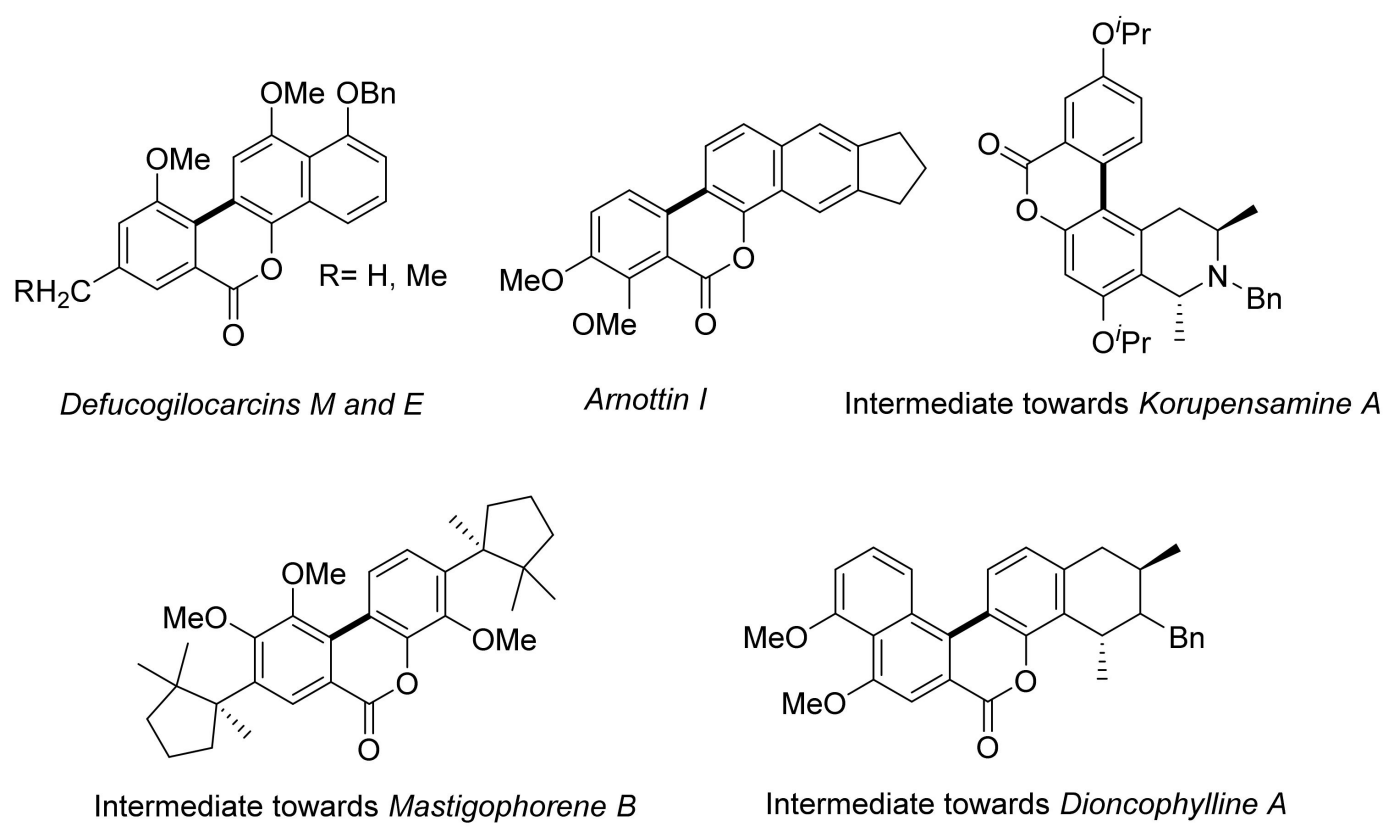
Natural products and synthetic precursors obtained via direct arylation.

Direct arylation has been a tool for the construction of several natural products, polycyclic aromatic hydrocarbons (PAHs) and other chemically relevant compounds [88,89,90]. Indeed, the structure of several biologically relevant lactones prepared by direct arylation, including *Defucogilocarcins M* and *E* [91,92], and intermediates for the syntheses of *Dioncophylline A* and *Mastigophorene B* [93,94,95], are shown in Figure 2. Structurally related *Arnottin I* was prepared by Harayama and co-workers by direct arylation using Pd(OAc)_2_ (10 mol%) [96]. The preparation of several korupensamines, i.e., a family of naphthyltetrahydroisoquinoline alkaloids with antimalarial activity, was reported by Bringmann. An intramolecular direct arylation provided the lactone-bridged biaryl displayed in Figure 2, which was atroposelectively cleaved. Arylation employing Herrmann’s catalyst [97] provided a good yield of the required biaryl intermediate, whereas poor results were obtained by using Pd(OAc)_2_/PPh_3_. Accordingly, palladacycles can be useful, more efficient palladium sources for direct arylation [98].

**Scheme 3:**
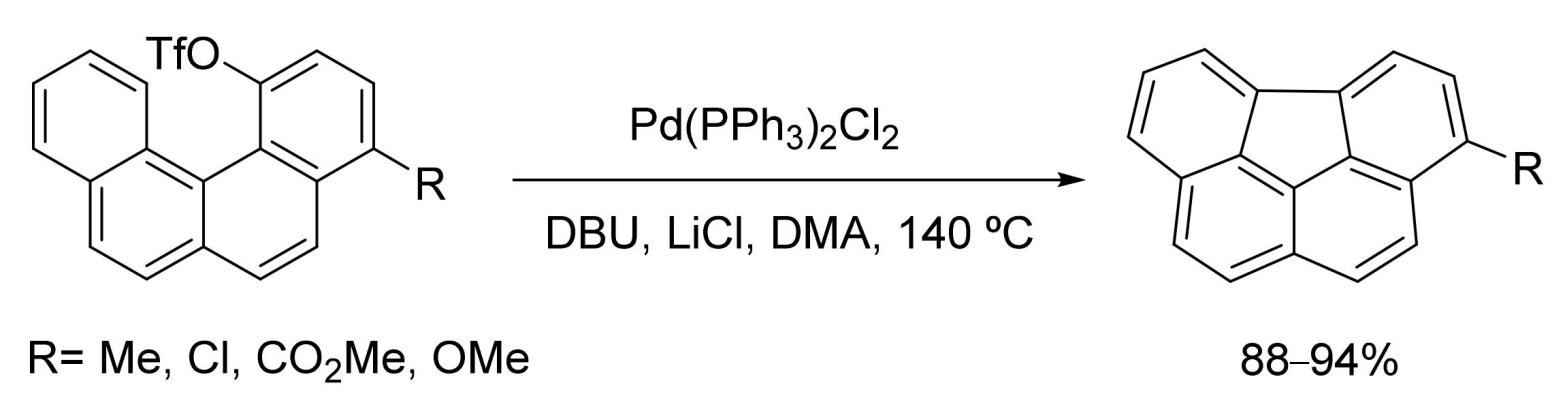
Intramolecular direct arylation for the synthesis of PAHs.

Bowl-shaped PAHs are typical synthetic precursors for the access to fullerenes. These fullerene fragments have been prepared in moderate to poor yields by flash vacuum pyrolysis. Moreover, as a result of the harsh reaction conditions needed, only a limited substrate scope is achieved, and this method is difficult to scale up. Following a pioneering report by Rice and co-workers [99], a number of PAHs including bowl-shaped fullerene fragments have been successfully synthesized by the intramolecular direct arylation of *o*-functionalized biaryl and benzophenanthrene derivatives. High yields and a good tolerance of functional groups were achieved (Scheme 3) [100,101].

Moulton and Shaw [102] reported the first examples of pincer complexes in 1976. High thermal, air and moisture stability were exhibited by palladium pincer complexes due to the tight coordination of the tridentate ligand to palladium. Although initially most of these complexes were symmetrical, non-palindromic ligands with hard and soft donor atoms have also been incorporated, thus providing a whole variety of structural designs. Depending on the latter structural features, interaction with substrates and/or the stabilization of reaction intermediates can be facilitated [103,104]. In fact, an increasing number of reports on the application of these terdentate complexes as catalysts or pre-catalysts for a number of synthetic transformations have been published, including recent papers on cross-coupling reactions catalyzed by pincer compounds [105,106,107,108,109,110,111,112,113,114,115,116,117,118,119,120]. In this regard, inter- and intramolecular direct (hetero)arylations have been carried out in the presence of several pincer complexes. This review will cover the literature on the use of palladium pincer complexes for the C–H functionalization of (hetero)arenes with (hetero)aryl halides. A brief summary of the reaction scope, and in some cases, proposals on the role of the pincer complex, will be discussed.

## 2. Palladium(II) Complexes with Phosphine-Containing Pincer Ligands 

**Scheme 4:**
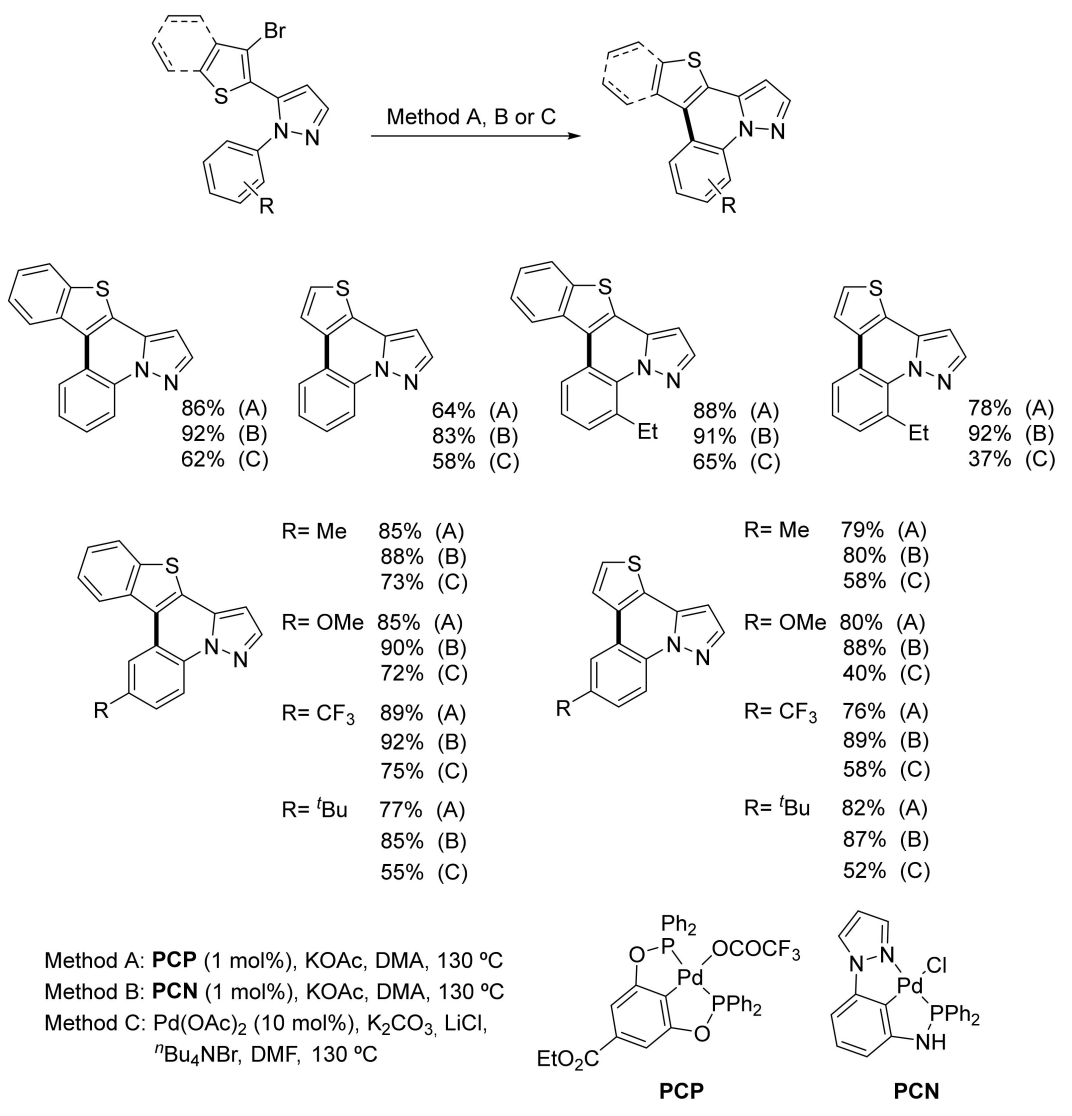
Synthesis of pyrazolo(benzo)thienoquinolines by direct heteroarylation.

Two palladium PCP and PCN complexes were tested as catalysts for the direct access to pyrazolo(benzo)thienoquinolines. This approach involved the intramolecular heteroarylation of 1-aryl-5-(benzo)thienylpyrazoles. The authors confirmed the excellent performance of the above complexes in comparison with commercially available Pd(OAc)_2_. Indeed, good to excellent yields for the target tetra- and pentacyclic compounds were obtained by using a relatively low amount (1 mol%) of phosphinite- and phosphinoamide-based PCP and PCN complexes. As for Pd(OAc)_2_, a significantly higher 10 mol% was required to catalyze the same reaction under Jeffrey’s ligand-free conditions, and even then, the yields obtained were lower in all cases. However, no clear differences were found between the catalytic ability of symmetric **PCP** and non-symmetric **PCN** complexes (Scheme 4). In addition to this palladium-catalyzed intramolecular heteroarylation, the authors also reported the intermolecular regioselective C-5 arylation of simple 1-substituted thiophenes with an equimolecular amount of bromobenzene under similar conditions [121].

**Scheme 5:**
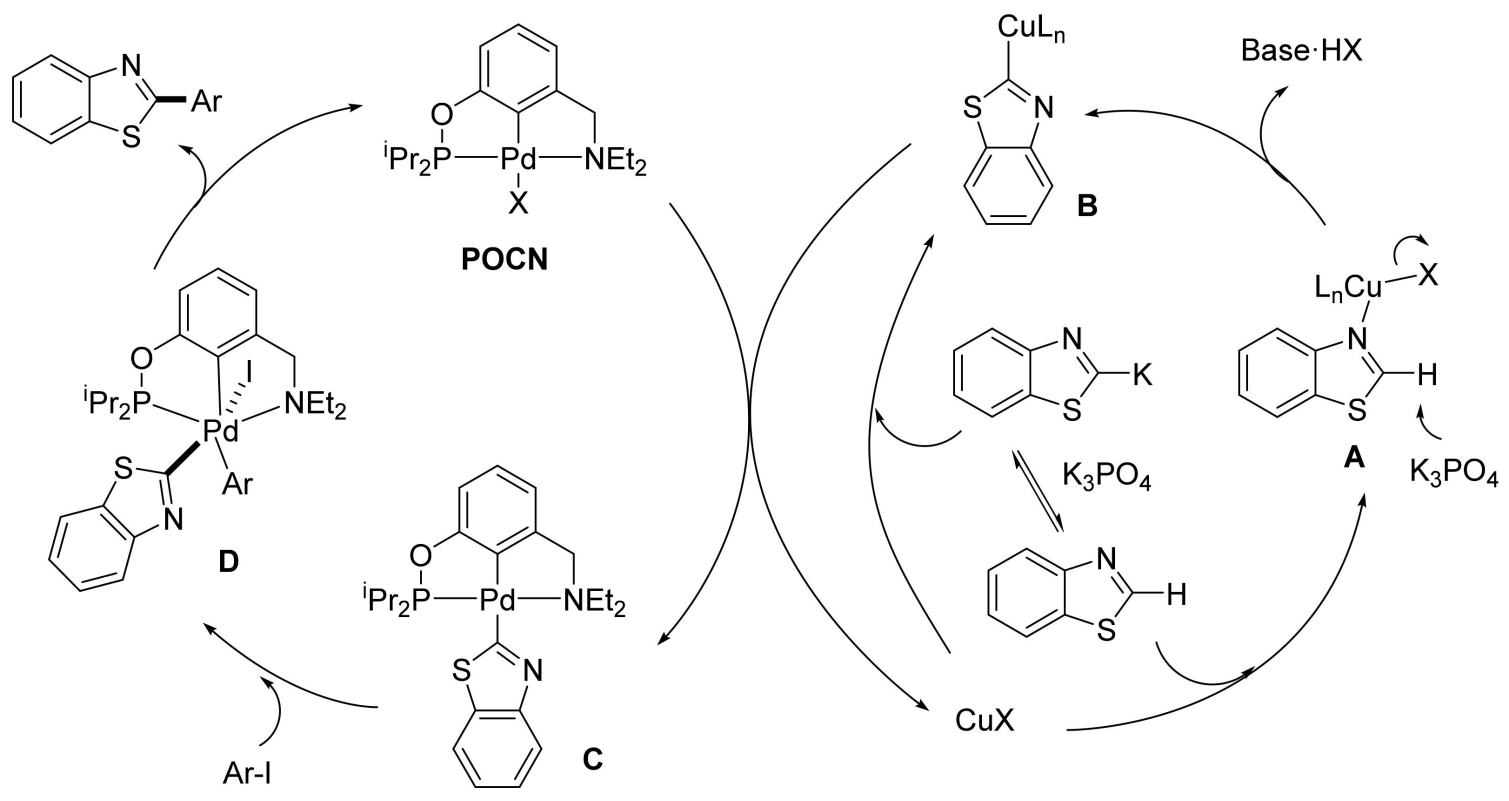
Mechanistic proposal for the direct heteroarylation of azoles.

Punji and coworkers reported the intermolecular direct C-2 arylation of benzothiazoles with aryl iodides in the presence of a palladium PCN pincer complex (**POCN**). 5-Aryloxazoles were also regioselectively arylated at the C-2 position under the same reaction conditions, which involved the use of cesium carbonate as a base and a slight excess of the iodoarene (1.5 equiv.) in DMF at 120 °C. Catalyst loading was optimized at 0.5 mol%, although it was necessary to add CuI (5 mol%) as a co-catalyst. An extensive study on the mechanism of the reaction was carried out using benzothiazole as a model substrate. After observing that the addition of ^n^Bu_4_NBr, a known stabilizer of palladium nanoparticles, did not have a beneficial effect on the reaction outcome, and noticing the results of some poisoning assays and of ^31^P-NMR monitorization, the authors proposed that, in contrast to previous reports on direct arylation reactions, a Pd(II)–Pd(IV)–Pd(II) pathway could be responsible for the presented arylation. As a result, the authors suggested that the catalytic cycle begins with coordination of benzothiazole with CuX to generate copper complex **A**, which turns, after H-2 deprotonation, into species **B**. Alternatively, **B** could be formed by an initial deprotonation followed by interaction with CuI. Copper-benzothiazolyl complex **B** would then promote transmetalation with palladium pincer complex PCN (**POCN**) leading to complex **C**, which was isolated. Oxidation addition of **C** with the aryl iodide would generate octahedral Pd(IV) complex **D** which, upon reductive elimination, would provide the product as well as the initial PCN complex **POCN** (Scheme 5). Some of the suggested intermediates were isolated and submitted to reaction conditions, providing the expected arylated products. Moreover, these results were corroborated by the kinetic studies and DFT calculations performed by the authors [122,123].

**Scheme 6:**
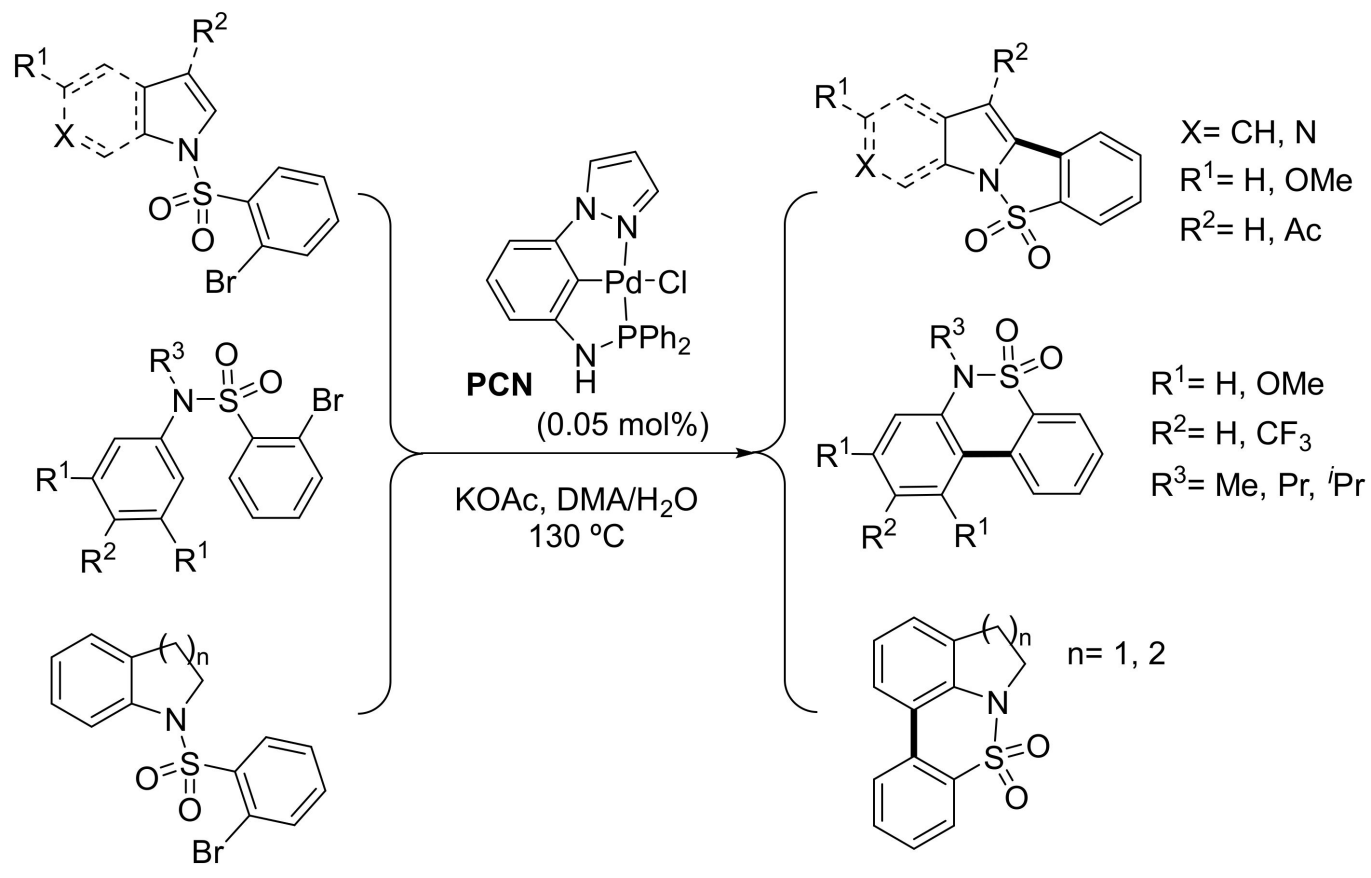
Intramolecular arylation of *o*-bromobenzenesulfonamides.

As a follow-up research on the results from their previous work on direct heteroarylation [121], in 2015, SanMartin’s group reported the intramolecular direct arylation of amides and sulfonamides in the presence of a PCN palladium pincer complex. The addition of a small amount of water was the key for regioselective access to a wide number of structurally diverse phenanthridinone derivatives using an even smaller amount of catalyst (0.05 mol%) of the phosphinoamide-based PCN palladium pincer depicted in Scheme 6. In addition, benzoisothiazoloindole 5,5-dioxides, benzothiazinoindole 7,7-dioxides, benzopyrroloisothiazole 5,5-dioxides and other biologically relevant sulfur heterocycles were prepared from 2-bromobencenesulfonamides under the same reaction conditions (Scheme 3) [124]. A major advantage of the presented method was the low amount of trace palladium impurities in the final products (0.29 ppm, measured by ICP-MS), certainly due to the small catalyst amount employed. The presence of metal traces in final products is a serious concern for the pharmaceutical industry, with increasingly stricter regulations that limit metal contents to 1 ppm or even less depending on the drug administration route (oral, parenteral, nasal, etc.). In many cases, costly and tedious purification steps are required to remove these toxic contaminants [125]. Wang et al. synthesized a phosphinite-based PCN palladium pincer complex comprising a benzimidazole unit and used it for the direct arylation of (benzo)thiazole and benzoxazole with aryl iodides. As in the paper by Punji [122], CuI was added as a co-catalyst. Using cesium carbonate as a base, they were able to reduce the amount of the palladium catalyst to 0.25 mol%. However, in some cases, 0.5 mol% of the pincer complex and 2.5–5 mol% of CuI were required to attain reasonable yields. A Pd(II)–Pd(IV)–Pd(II) pathway was also proposed to explain the role of the above pincer complex in the arylation reaction [126].

## 3. NHC Containing Pincer Complexes

**Scheme 7:**
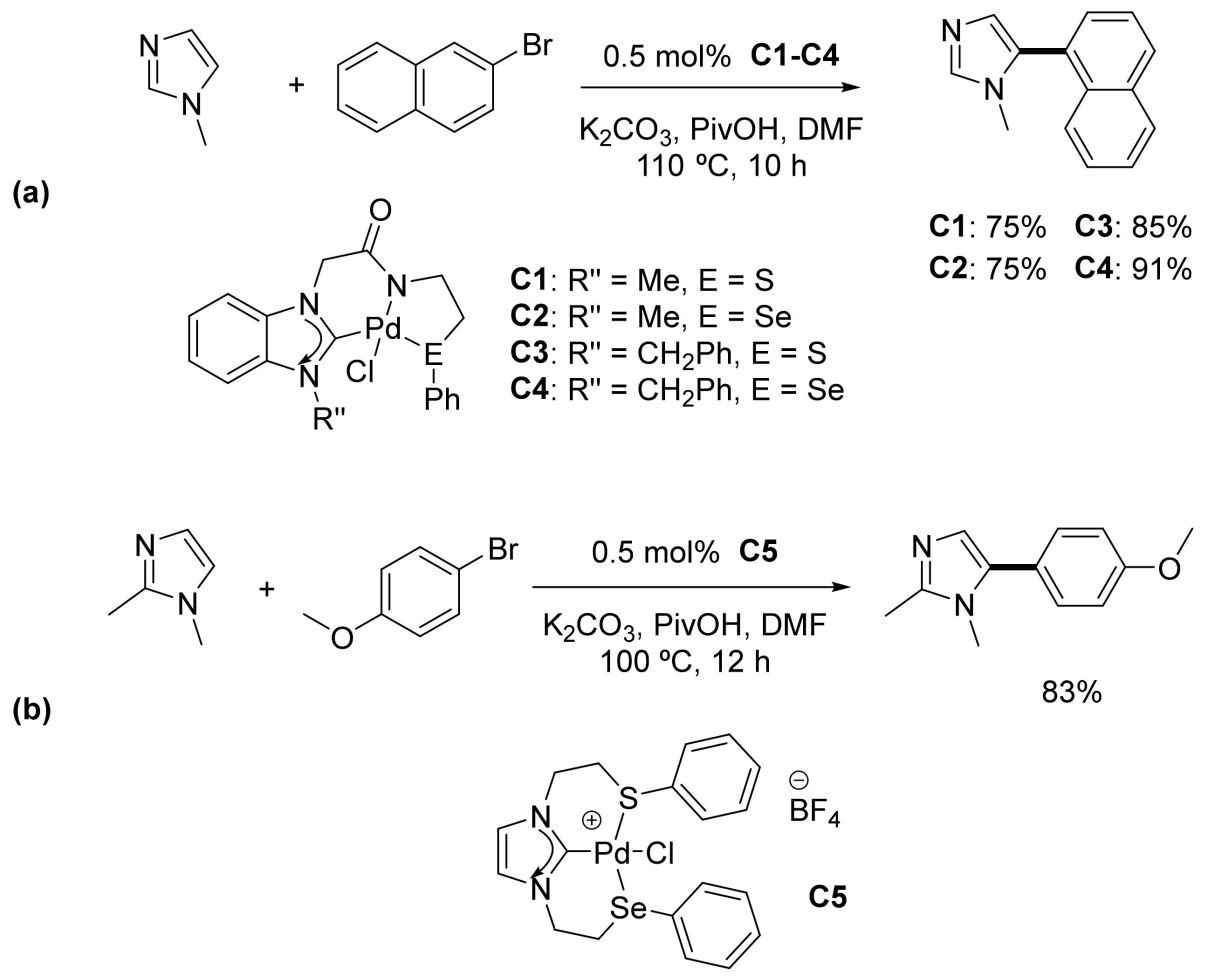
Regioselective C-5 arylation of 1-methyl- and 1,2-dimethylimidazole derivatives as described by Singh (**a**) and by Joshi (**b**).

*N*-heterocyclic carbenes (NHC) were introduced in organometallic chemistry by Öfele, who reported the first example back in 1968 [127]. Carbene moieties are usually incorporated in bi- and tridentate ligands due to the high stability they can provide to metal complexes [128,129]. Singh’s group reported the first use of palladium pincer complexes containing NHC moieties as catalysts for direct arylation reactions. After preparing a series of non-palindromic CNS and CNSe complexes (**C1**–**C4**) derived from chalcogenated acetamide-functionalized benzimidazolium salts, their catalytic performance in the regioselective C-5 arylation of 1-methyl- and 1,2-dimethylimidazoles with aryl halides under aerobic conditions was examined. A substoichiometric amount of pivalic acid (30 mol%) turned out to be crucial for the reaction outcome. In this regard, the authors proposed that pivalic acid generates coordinatively unsaturated Pd as its proton neutralizes the anionic nitrogen of the amidate fragment, thus helping in the cleavage of the Pd–N bond. That behavior would be consistent with a concerted metalation–deprotonation (CMD) pathway. Benzoic acid was also assayed, providing negligible results. The authors rationalize this outcome considering the higher acidity of benzoic acid, which would stabilize the reaction intermediates so that no further catalysis can occur. As for pincer complexes, 0.5 mol% of catalysts **C1**–**C4** was chosen as optimal for the reaction scope, illustrated by the arylation with sterically hindered 1-bromonaphthalene, as depicted in Scheme 7a. The regioisomeric identity of some of the arylated products was additionally determined by single-crystal X-ray diffraction analysis. Heteroarylation with 3-bromopyridine and 3-bromoquinoline was also carried out under the same conditions. Minor side-products from the C-4 arylation of imidazole and homocoupling of aryl bromides were also detected. 4-Chlorobenzaldehyde and 4-chlorobenzonitrile were also successfully used as arylating agents, although a higher amount of the catalyst (1 mol%) and longer reaction times (20–24 h) were required, probably due to a more difficult oxidative addition step. In addition, the catalytic life of **C1**–**C4** was also tested by recycling or reusing these complexes for six runs. Good yields were obtained in all cases, although a steady decrease was observed in every consecutive run [130]. Very similar reaction conditions (K_2_CO_3_, PivOH, DMA, 110 °C) were used by Joshi and co-workers to effect the direct C-5 arylation of imidazole derivatives with aryl bromides in the presence of an SCSe complex (**C5**), where the NHC moiety occupied the central position of the tridentate ligand (Scheme 7b). Arylation with 4-nitrochlorobenzene was also carried out, although a significant decrease in the reaction yield was observed. In addition, catalyst **C5** demonstrated remarkable recyclability up to five consecutive cycles [131]. Following Finke’s report on procedures to distinguish between homogeneous and heterogeneous palladium catalysts [132], both research groups performed poisoning experiments with Hg and PPh_3_ (Pd/Hg (1/400), 5 mol % of PPh_3_). Palladium nanoparticles and other palladium (0) species amalgamate with mercury so that a noticeable drop in the conversion yield is observed (mercury drop test). However, no inhibition was observed for the above direct arylation reactions after adding overstoichiometric amounts of these poisoning agents. Considering the results from these poisoning assays and the recyclability exhibited by their pincer complexes, the authors suggested that the catalysis was homogeneous in nature [130,131].

**Scheme 8:**
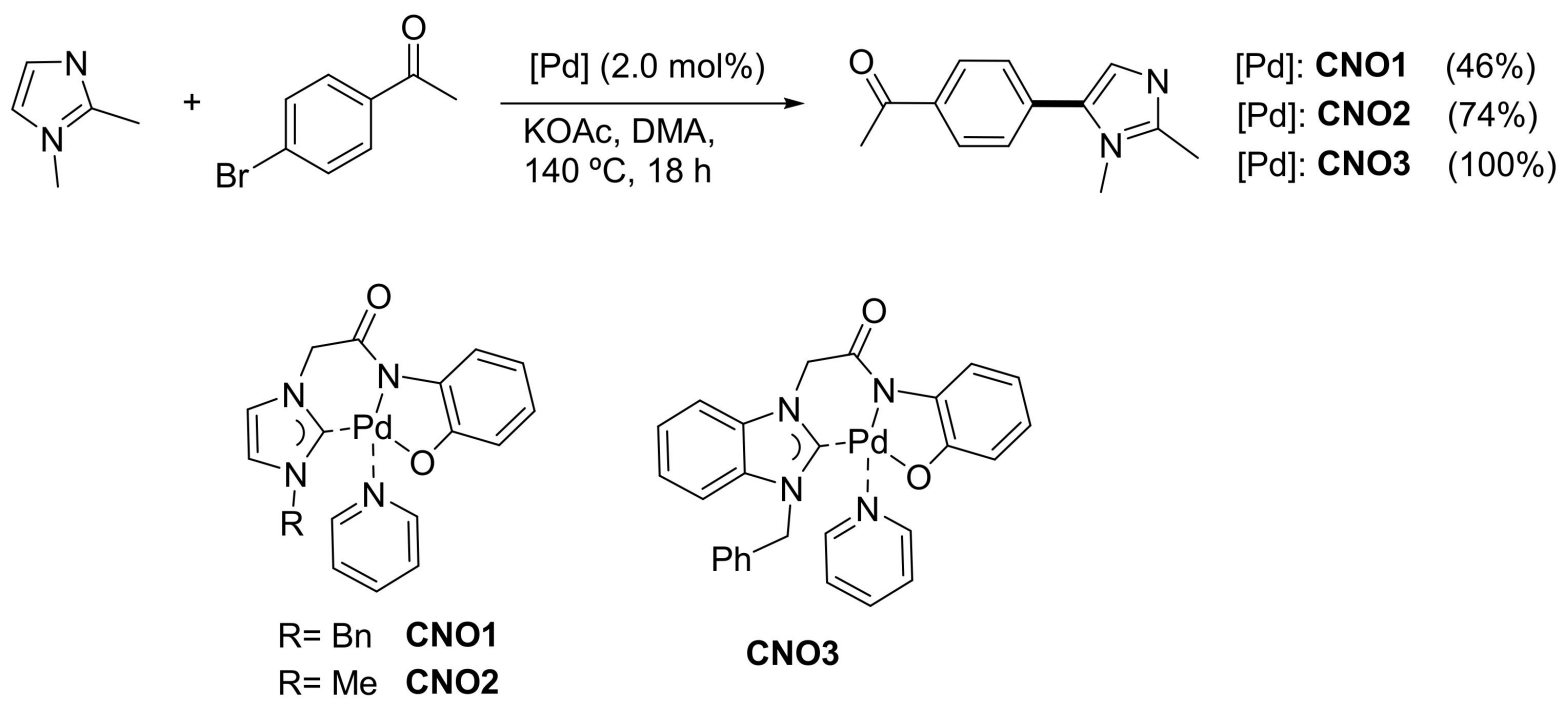
C-5 arylation of 1,2-dimethylimidazole in the presence of CNO pincer complexes.

The selective arylation of 1,2-dimethylimidazole and imidazo[1, 2-*a*]pyridine derivatives with bromoarenes in the presence of 2 mol% of CNO palladium(II) complexes containing NHC moieties (**CNO1**–**CNO3**) was studied by Lee and co-workers (Scheme 8). They also compared their catalytic activity with that of several palladium sources and ligands (PdCl_2_, Pd(OAc)_2_, Pd(OAc)_2_/PCy_3_, etc.) and found that their CNO complex was less active than their previously reported Pd(0) complex featuring bidentate NHC and PPh_2_ moieties [133], which could catalyze arylation with chloroarenes. PEPPSI precatalyst Pd(IPr)(3-ClPy)Cl_2_ [134] also provided the product from the benchmark reaction, the C-5 arylation of 1,2-dimethylimidazole and 4-bromoacetophenone, with equal efficiency. As in many other reports on direct arylation processes, DMA was the solvent of choice, and a significant decrease in the yields was observed when switching to DMF, THF or toluene. Except for 2-bromobenzonitrile and 2-bromotoluene, all the sterically hindered bromoarenes failed to furnish the desired product from 1,2-dimethylimidazole at 140 °C.

**Scheme 9:**
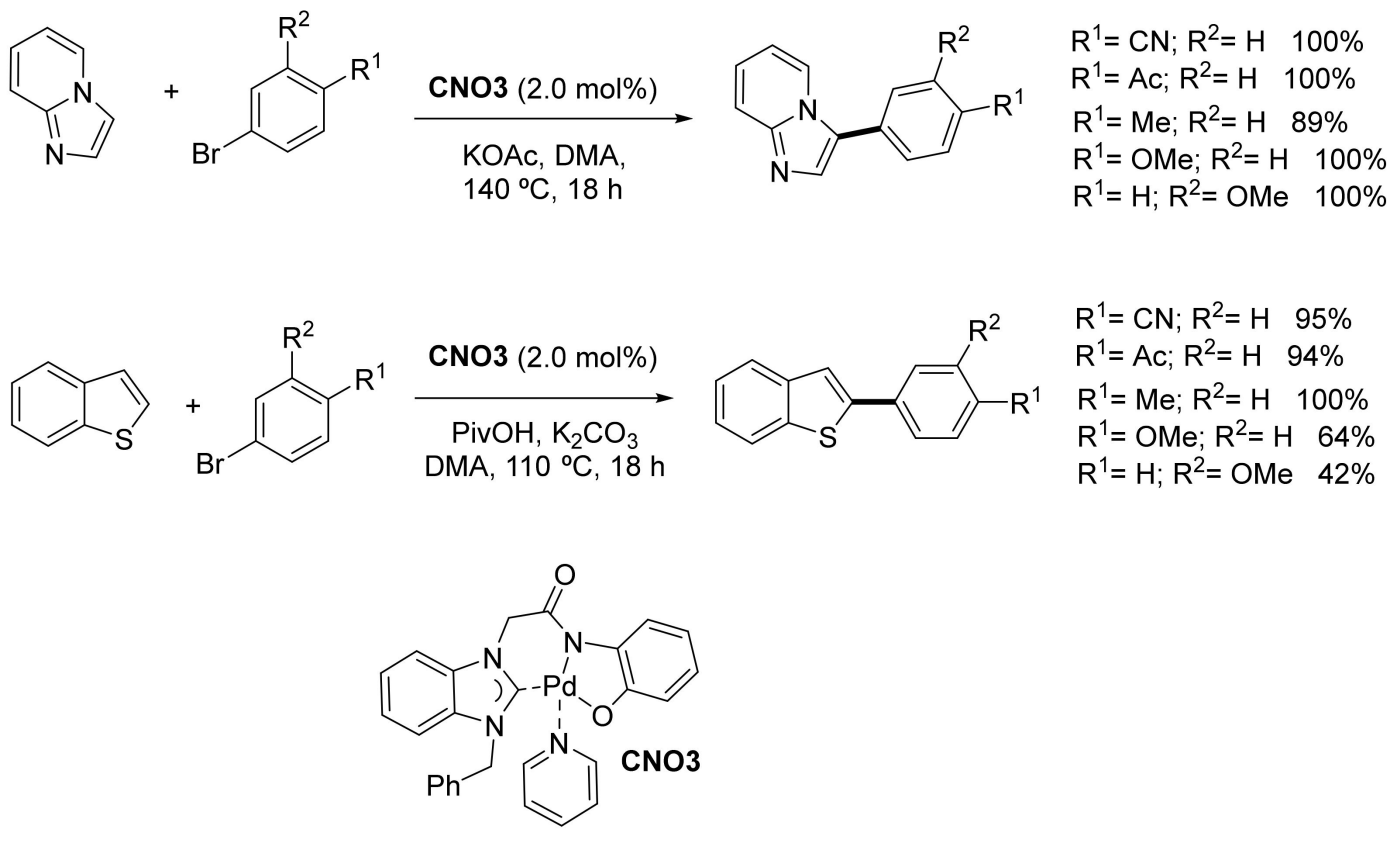
Direct arylation of imidazo[1,2-*a*]pyridine and benzothiophene.

In the same paper, imidazo[1,2-*a*]pyridine was reacted with several bromoarenes bearing electron-donating and electron-withdrawing substituents to provide the corresponding 3-arylated compounds with good to excellent yields. In contrast to 1,2-dimethylimidazole, similar yields were achieved for imidazo[1,2-*a*]pyridine when the reaction was carried out under argon and under air. 2-Arylbenzothiophenes were also obtained by reactions between thiophene and arylbromides, and in this case, at lower temperature (110 °C, Scheme 9). Gram-scale reactions (10 mmol) between 1,2-dimethylimidazole and 4-bromobenzonitrile, and between imidazo[1.2-*a*]pyridine and 4-methoxybromobenzene, provided the corresponding arylated compounds in 70% and 66% yields, respectively.

**Scheme 10:**
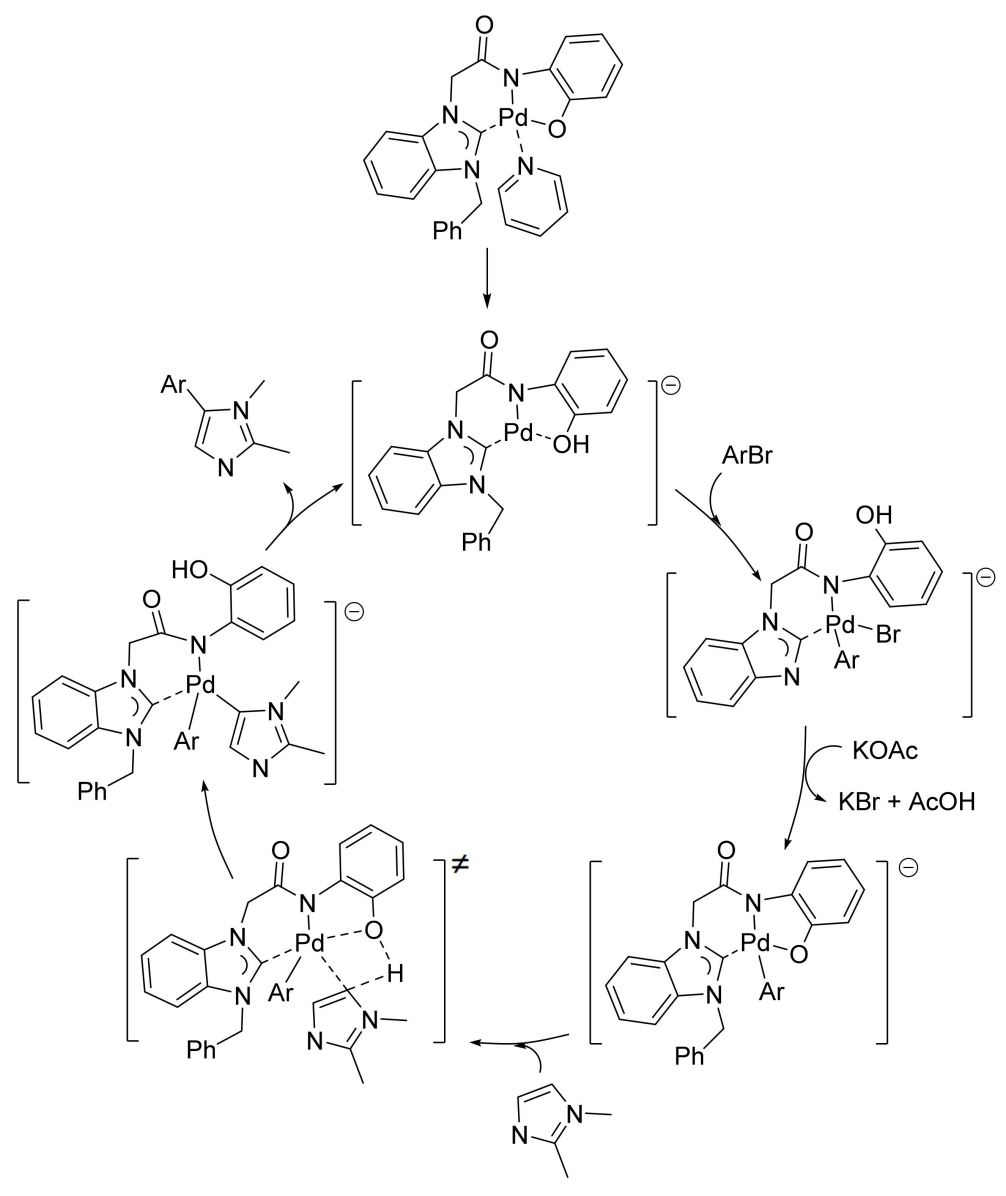
Mechanism proposed by Lee and co-workers for the C-5 arylation of 1,2-dimethylimidazole.

The authors also carried out competitive reactions using an equimolecular mixture of 1,2-dimethylimidazole and imidazopyridine and the same bromoarene. After observing that electron-poor imidazopyridine prevailed over electron-rich 1,2-dimethylimidazole (3-arylimidazopyridine was mainly obtained when using 4-bromoanisole, and exclusively isolated when 4-bromoacetophenone was the arylating agent), they suggested that the arylation proceeds via a Pd(II)–Pd(0)–Pd(II) mechanism based on a concerted metalation–deprotonation (CMD) step (Scheme 10). On account of the electron-donating nature of the 1,2-dimethylimidazole unit and the electron-withdrawing character of the imidazo[1.2-*a*]pyridine core, they synthesized several push–pull chromophores that exhibited a deep blue photoluminescence with moderate quantum efficiency on a large scale, and twisted the intramolecular charge transfer excited state [135].

## 4. Other Pincer Complexes

**Scheme 11:**
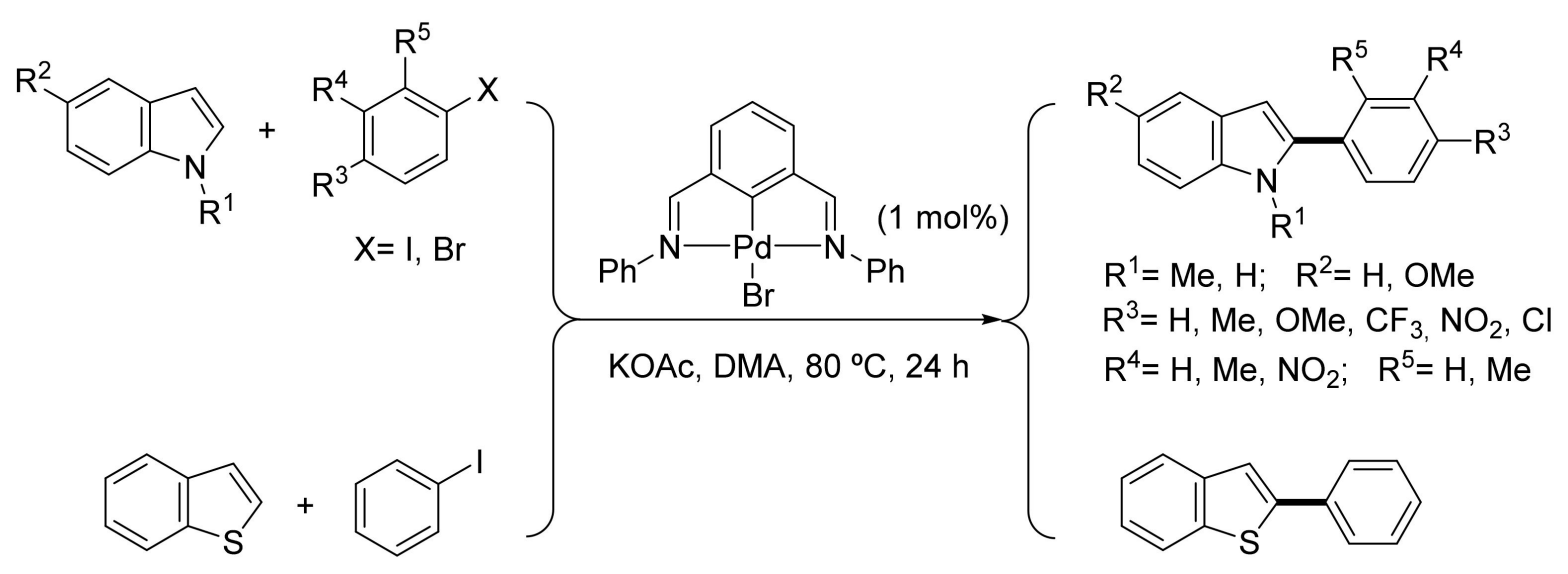
NCN palladium pincer complex as an active catalyst for the regioselective arylation of indoles.

Direct arylation has been also reported in the presence of palladium pincer complexes lacking phosphine of NHC moieties. Cai and co-workers prepared a symmetric Schiff-based NCN complex and used it for the selective direct arylation of *N*-methylindoles at C-2. After some preliminary assays with *N*-methyl-1*H*-indole and iodobenzene as model substrates, significantly lower yields (22–46%) were obtained using Pd(OAc)_2_ or Pd_2_dba_3_ (5 mol%) than with their NCN complex (1 mol%). Regarding regioselectivity, reactions carried out in dimethylacetamide at 80 °C provided 2-phenylated product as the only regioisomer, whereas significant amounts of the 3-phenylated product were observed when other solvents (NMP, DMF, AcOH) were used. A further decrease in the catalyst amount to 0.5 mol% diminished the yield to 77%; therefore, the optimized conditions displayed in Scheme 11 (NCN 1 mol%, KOAc, DMA, 80 °C) were applied to a number of aryl iodides, obtaining the corresponding *N*-methyl-2-arylindoles in moderate to good yields. However, only bromoarenes bearing electron-withdrawing substituents provided acceptable results. Interestingly, 1*H*-indole and benzothiophene were also regioselectively phenylated with moderate yields under the same conditions. Observation of palladium-black and complete inhibition upon the addition of mercury (mercury drop test) led the authors to propose that their NCN pincer complex is a precatalyst or reservoir of Pd(0) species [136].

**Scheme 12:**
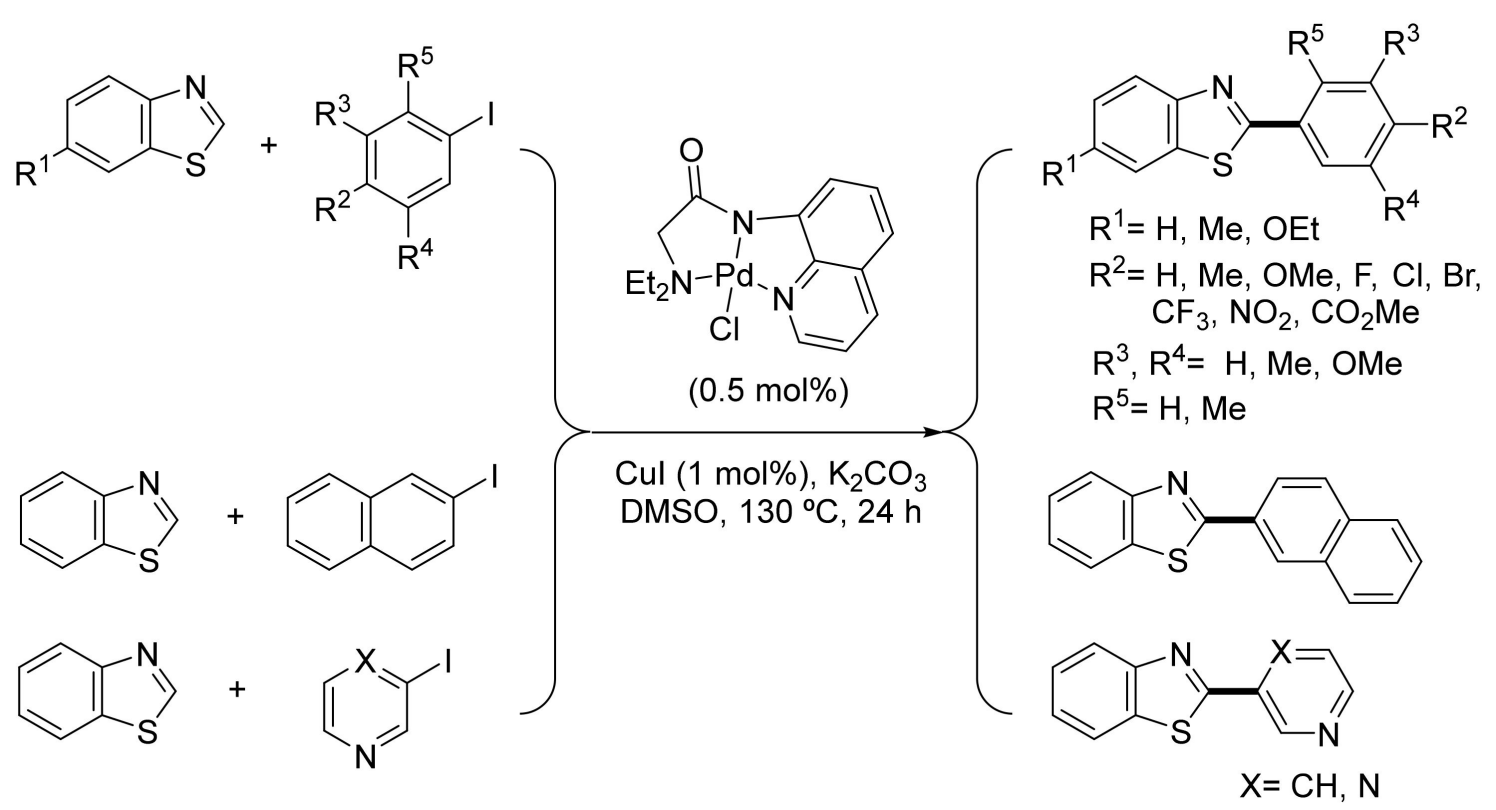
Direct arylation with aryl iodides in the presence of a NNN palladium pincer complex and CuI.

In addition to their previous work on a PCN complex [122,123], Punji’s group prepared several phosphine-free NNN palladium pincer complexes containing a (quinolinyl)amido moiety. One of them turned out to be an efficient catalyst for the direct arylation of benzothiazoles with aryl and heteroaryl iodides in the presence of CuI (1 mol%, Scheme 12). After removal of the arylation product by vacuum distillation and addition of the reagents and solvent, this catalyst was recycled up to five times with a minor decrease in the reaction yield. A hot filtration experiment was performed after the initial heating (30 min, GC yield 34%) to remove all the heterogeneous particles that might account for the slight decrease in the yield observed when adding overstoichiometric amounts of mercury. The reaction was continued upon adding fresh base (K_2_CO_3_), and the arylation product was obtained with good yield (88%). On the basis of the results from these two experiments and other mechanistic investigations (kinetic plot, observation of the reactivity order for several aryl iodides, MALDI-TOF-MS analysis of the reaction mixture, etc.), the authors proposed a catalytic cycle akin to that displayed in Scheme 5 [137].

**Scheme 13:**
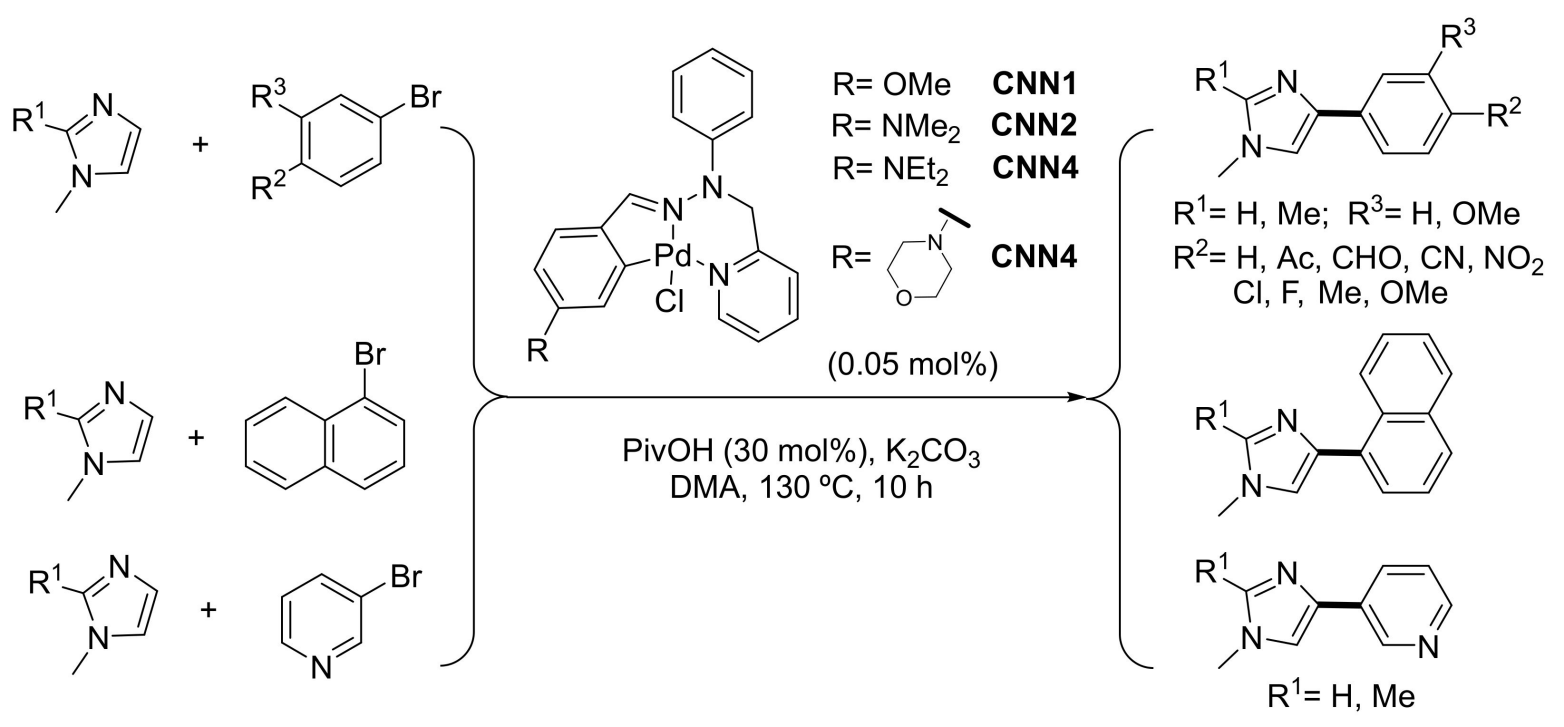
C-5 arylation with bromoarenes in the presence of **CNN1**–**CNN4** palladium pincer complexes.

Maji et al. prepared two ferrocene-based palladium CNN pincer complexes by palladation of the ligands obtained by a condensation of ferrocenecarboxaldehyde with 2-(1-phenylhydrazinyl)pyridine or 2-((1-phenylhydrazinyl)methyl)pyridine. After performing Suzuki–Miyaura biaryl couplings with aryl chlorides, the efficient C-5 arylation of 4-methylthiazole and the C-4 arylation of 3,5-isoxazole with aryl bromides were explored. As for the Suzuki–Miyaura couplings, 0.1 mol% of their CNN complex was enough to catalyze the direct arylation reactions. Good yields were obtained regardless of the electronic nature of the bromoarene. Palladium nanoparticles, generated in situ by decomposition of these pincer complexes, were thought by the authors to be the real catalyst species through a Pd(0)-Pd(II) cycle [138]. A year later, they reported the preparation of four structurally related CNN complexes by the condensation of benzaldehyde derivatives and 2-(1–2-((1-phenylhydrazinyl)methyl)pyridine followed by palladation with Na_2_PdCl_4_. Optimization of the model reaction, the arylation of 1-methyl-1*H*-imidazole with 4-bromobenzaldehyde, was carried out with one of the four tricoordinated complexes. Then, the scope of the reaction was expanded and 1-methyl- and 1,2-dimethylimidazole were regioselectively arylated (C-5) with bromoarenes by using 5 × 10^−2^ mol% of this palladium source (Scheme 13).

**Scheme 14:**
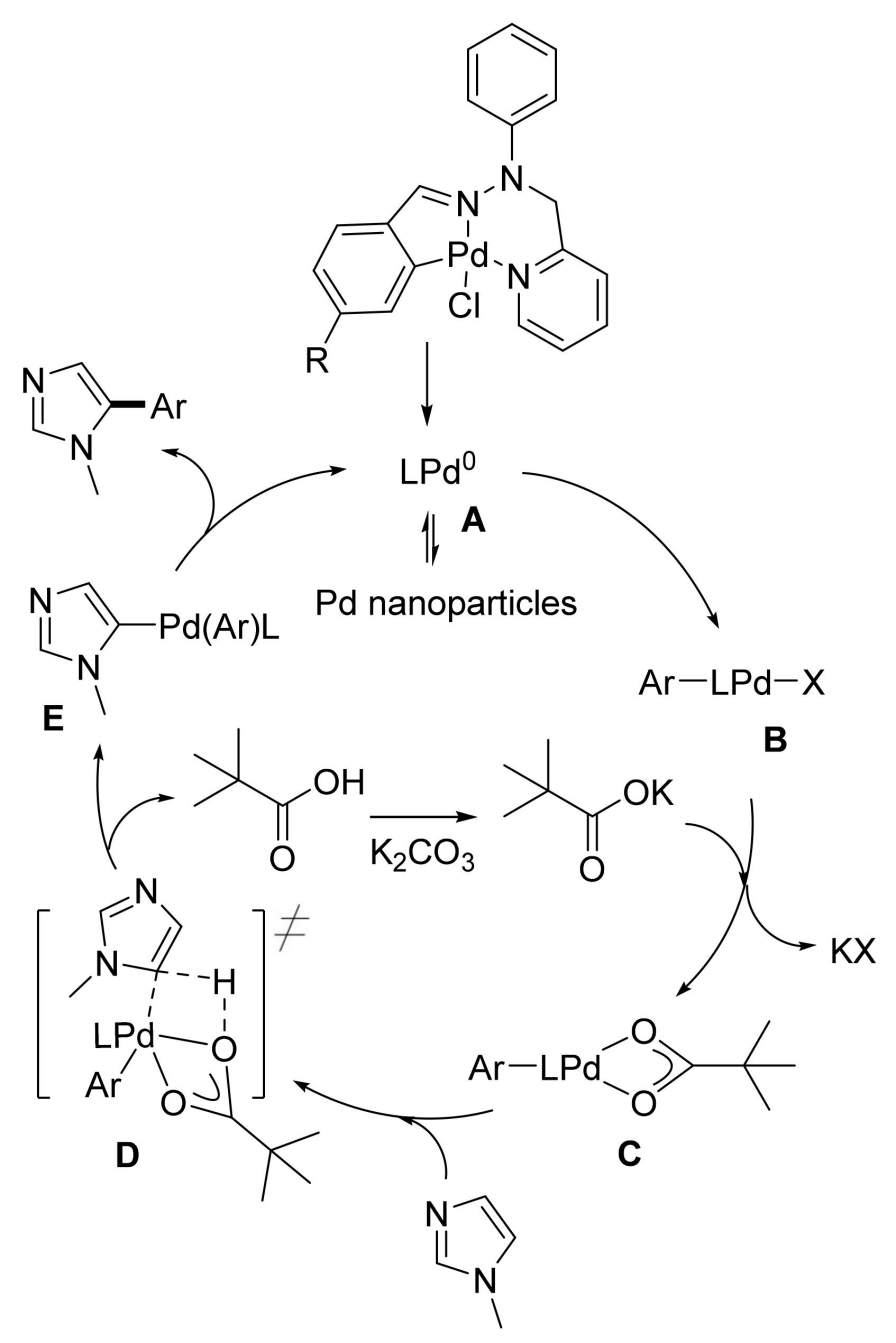
Possible mechanism for the direct regioselective arylation of 1-methylimidazole derivatives.

The procedure was also useful for the arylation of the same azoles with aryl chlorides in the presence of another of the four CNN complexes, although a slight increase in the amount of the later palladacycle was required (0.1 mol%). In order to explain the reaction mechanism, the authors proposed the catalytic cycle displayed in Scheme 14. After the in situ generation of palladium(0) species **A**, oxidative addition with the aryl halide provided intermediate **B**, which underwent a ligand exchange with potassium pivalate to form intermediate **C**. Interaction with methylimidazole generated intermediate **E** via a CMD transition state (**D**), and after a reductive elimination step, the arylated product was released along with the Pd(0) species [139].

**Scheme 15:**
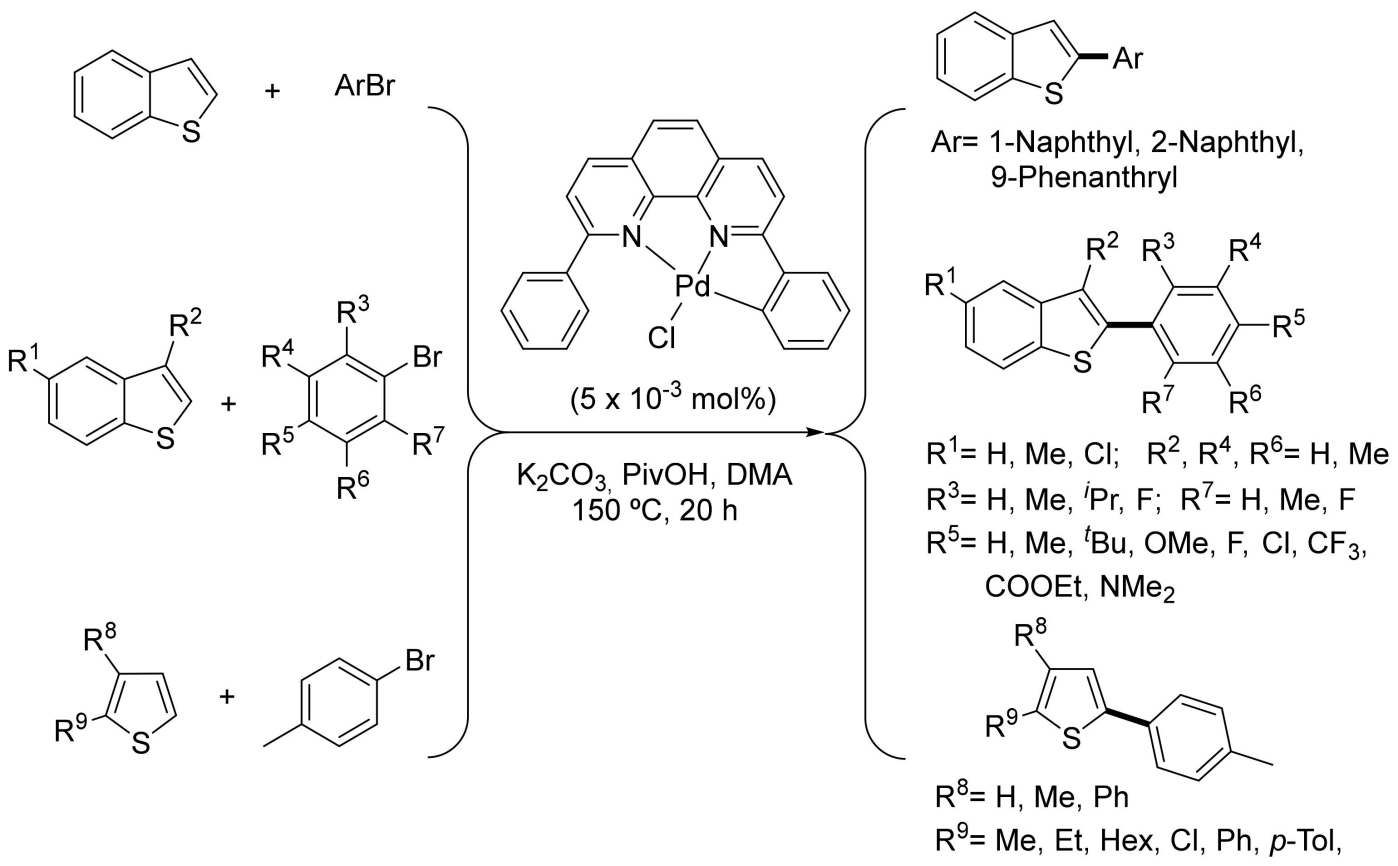
Direct arylation of thiophene derivatives in the presence of a CNN palladium pincer complex.

Following their research on Heck and Hiyama reactions, Uozumi’s group described the use of infinitesimal amounts (5 × 10^−3^ mol%) of a phenanthroline-based CNN pincer for the direct arylation of (benzo)thiophene derivatives with aryl bromides. Benzothiophenes were arylated with electronically dissimilar and sterically hindered bromoarenes. Regioselective C-5 arylation was observed for 2-substituted and 2,3-disubstituted thiophenes (Scheme 15). Regarding the role of the above CNN complex, palladium nanoparticles (average diameter 3.2 nm) were detected by TEM (transmission electron microscopy) after completion of the reaction. Accordingly, the authors proposed that monomeric palladium(0) species released from the pincer complex were responsible for the catalytic activity observed [140].

## 5. Conclusions

Direct arylation has been consolidated as an advantageous alternative to cross-coupling reactions involving transmetallating agents. In this regard, the use of palladium pincer complexes as (pre)catalysts for this reaction has attracted much attention because of the lower catalyst amounts required. However, depending on the coupling partners and the complex employed, it is not clear if such efficiency is due to a steady release of palladium(0) species (e.g., palladium nanoparticles) from the complex, to a Pd(II)–Pd(IV) catalytic cycle or to a cocktail of different mechanisms simultaneously taking place. Further research in this field will probably reveal the nature of the true catalysts and will expand the reaction scope by introducing new, more active pincer complexes. Finally, given the almost exclusive use of DMA and DMF as solvents in these reactions, safer reaction media would be also desirable.
